# Quantifying the effects of pollen nutrition on honey bee queen egg laying with a new laboratory system

**DOI:** 10.1371/journal.pone.0203444

**Published:** 2018-09-05

**Authors:** Julia D. Fine, Hagai Y. Shpigler, Allyson M. Ray, Nathanael J. Beach, Alison L. Sankey, Amy Cash-Ahmed, Zachary Y. Huang, Ieva Astrauskaite, Ran Chao, Huimin Zhao, Gene E. Robinson

**Affiliations:** 1 Carl R. Woese Institute for Genomic Biology, University of Illinois at Urbana-Champaign, Urbana, United States of America; 2 Department of Entomology, Michigan State University, East Lansing, United States of America; 3 Department of Chemical and Biomolecular Engineering, University of Illinois at Urbana-Champaign, Urbana, United States of America; 4 LifeFoundry, Inc., Champaign, United States of America; 5 Neuroscience Program, University of Illinois at Urbana-Champaign, Urbana, United States of America; 6 Department of Entomology, University of Illinois at Urbana-Champaign, Urbana, United States of America; University of North Carolina at Greensboro, UNITED STATES

## Abstract

Honey bee populations have been declining precipitously over the past decade, and multiple causative factors have been identified. Recent research indicates that these frequently co-occurring stressors interact, often in unpredictable ways, therefore it has become important to develop robust methods to assess their effects both in isolation and in combination. Most such efforts focus on honey bee workers, but the state of a colony also depends on the health and productivity of its queen. However, it is much more difficult to quantify the performance of queens relative to workers in the field, and there are no laboratory assays for queen performance. Here, we present a new system to monitor honey bee queen egg laying under laboratory conditions and report the results of experiments showing the effects of pollen nutrition on egg laying. These findings suggest that queen egg laying and worker physiology can be manipulated in this system through pollen nutrition, which is consistent with findings from field colonies. The results generated using this controlled, laboratory-based system suggest that worker physiology controls queen egg laying behavior. Additionally, the quantitative data generated in these experiments highlight the utility of the system for further use as a risk assessment tool.

## Introduction

Managed honey bee pollinators contribute an estimated 15 billion dollars yearly to the United States economy [1], and they have become crucial to ensuring food security for a growing population world wide [1–3]. However, declines in populations of pollinators, including honey bees, have caused concern, and researchers have now identified four key factors that negatively impact honey bee health: poor nutrition, exposure to pesticides, pathogens, and parasites [4–6]. Importantly, these stressors can interact in unpredictable ways [5]. The effects of these frequently co-occurring stressors highlight the need for robust methods to assess risks to honey bee health so that they can be mitigated.

To study the effects of individual and interacting stressors on the complex biological processes that occur within a honey bee colony, many researchers conduct field experiments with full size colonies or experimental colonies that have reduced populations or demographies [7–11]. These experiments produce environmentally relevant data pertaining to colony-level effects, but their designs are often time- and resource-intensive, with challenges in controlling for variables such as the effects of agrochemical residues persisting in wax comb [12] and in the surrounding foraging landscapes [13], the sources of nutrition available to the colony [14,15], bee genetic variation [16,17], exposure to new or worsening pathogen infections [11], and queen failure events [4]. Laboratory-based assays generally afford researchers more control over experimental parameters, but there is currently no laboratory-based method to screen for effects of stressors on queen egg laying.

Following successful insemination, the queen is the sole producer of the fertilized eggs necessary for maintaining the colony population [18]. Therefore, the queen’s health and productivity are critical to colony longevity [19]. Recently, high rates of queen failure and supercedure have been documented throughout the United States, and beekeepers have reported queen failure as a major cause of colony loss [20,21]. Several studies have indicated that queen failure could be due to agrochemical exposure [22,23] or stressful conditions during queen shipment affecting sperm viability in mated queens [24]. These observations highlight the need for controlled methods to study the effects of stressors on queen egg laying. However, the queen’s unique life history poses considerable challenges to researchers seeking to dissect the effects of stressors on queen fecundity from other colony level effects.

The honey bee queen relies on constant care and feeding by young worker bees [18]. This behavior, which is referred to as retinue behavior [25], is elicited as a response to a semiochemical blend produced by the queen known as queen pheromone [26,27]. Sustained queen egg laying is not known to occur in the absence of honey bee workers, therefore, egg-laying is the product of the coordinated efforts of both the queen and the workers in the colony. The relatively small number of quantitative studies of queen egg laying behaviors have been performed in full-sized or reduced population colonies, and researchers either cage the queen to restrict her egg laying [28,29] or use glass-walled observation hives to perform daily egg counts and assessments [30–32]. These mostly field-based studies have yielded valuable insights into the queen’s biology, life history, and the effects of stressors, but new laboratory-based methods that facilitate a higher degree of experimental control would speed the progress of queen health research. Additionally, federal regulators rely largely on laboratory-based tests to inform policy related to agrochemicals and honey bees [33], and the development of a laboratory-based screening system for effects of stressors on queens would help inform guidelines to protect pollinators.

Here, we describe a new laboratory-based system to quantitatively assess egg laying and use the system to examine the effects of pollen nutrition on queen fecundity. Within a colony, young adult nurse worker bees consume hive stored pollen, aka “bee bread,” to develop their hypopharyngeal glands (HPGs) [34]. These glands produce the proteinaceous secretions that nurse bees use to provision members of the hive including developing larvae and the queen [25,35,36]. Bee bread is made by mixing pollen, honey, and honey bee salivary secretions that contain bacteria commonly found in the honey bee digestive track [37–39]. Cage studies have shown that while honey bees can survive and develop their HPGs and other tissues when fed artificial sources of protein, consuming bee bread results in the most developed HPGs [40,41].

The relationship between HPG development and retinue behavior is not well established, but it is known that queens are typically provisioned by bees less than 12 days old [25]. Bees in this age range typically have highly developed HPGs [42]. Egg production in queens is correlated with vitellogenin production [43], and vitellogenin may also play a role in increasing queen longevity by reducing oxidative stress [44, 45]. Vitellogenin levels in newly eclosed, unmated queens increase after feeding [43], suggesting that nutrition can influence queen vitellogenin production. Similarly, while there is no established correlation between retinue behavior and egg laying, a correlation between queen feedings and egg laying has been documented [25], and diet quality has been shown to influence reproduction in honey bee and ant colonies [10,46]. We therefore hypothesized that HPG development and egg laying in this system can be manipulated through worker pollen feeding, and that feeding caged bees bee bread will result in higher egg laying and HPG development relative to commercially sourced pollen.

## Methods

### Bees

Wax comb frames containing capped worker brood (pupae and older larvae) were obtained from colonies maintained according to standard commercial methods at the Bee Research Facility at the University of Illinois Urbana-Champaign, Urbana, Illinois (UIUC) during May-September 2017. They were placed in a warm room (34.5°C) until adult eclosion. Newly eclosed worker bees were brushed off the frames and added to specially designed queen monitoring cages (QMCs; described below) by weight (10 g = approximately 100 bees). For each experiment, bees from 2–3 colonies were brushed from frames as they emerged and mixed before being added to cages to ensure a random distribution of bees from different colonies throughout the cages. Naturally mated queens of primarily Carniolan (subspecies) stock were purchased from Olivarez Honey Bees (Orland, CA). All queens used in these experiments were of the same genetic stock. Queen mortality was monitored and recorded throughout all experiments, and worker mortality was not quantitatively assessed.

### Cage design

**Fig 1:**
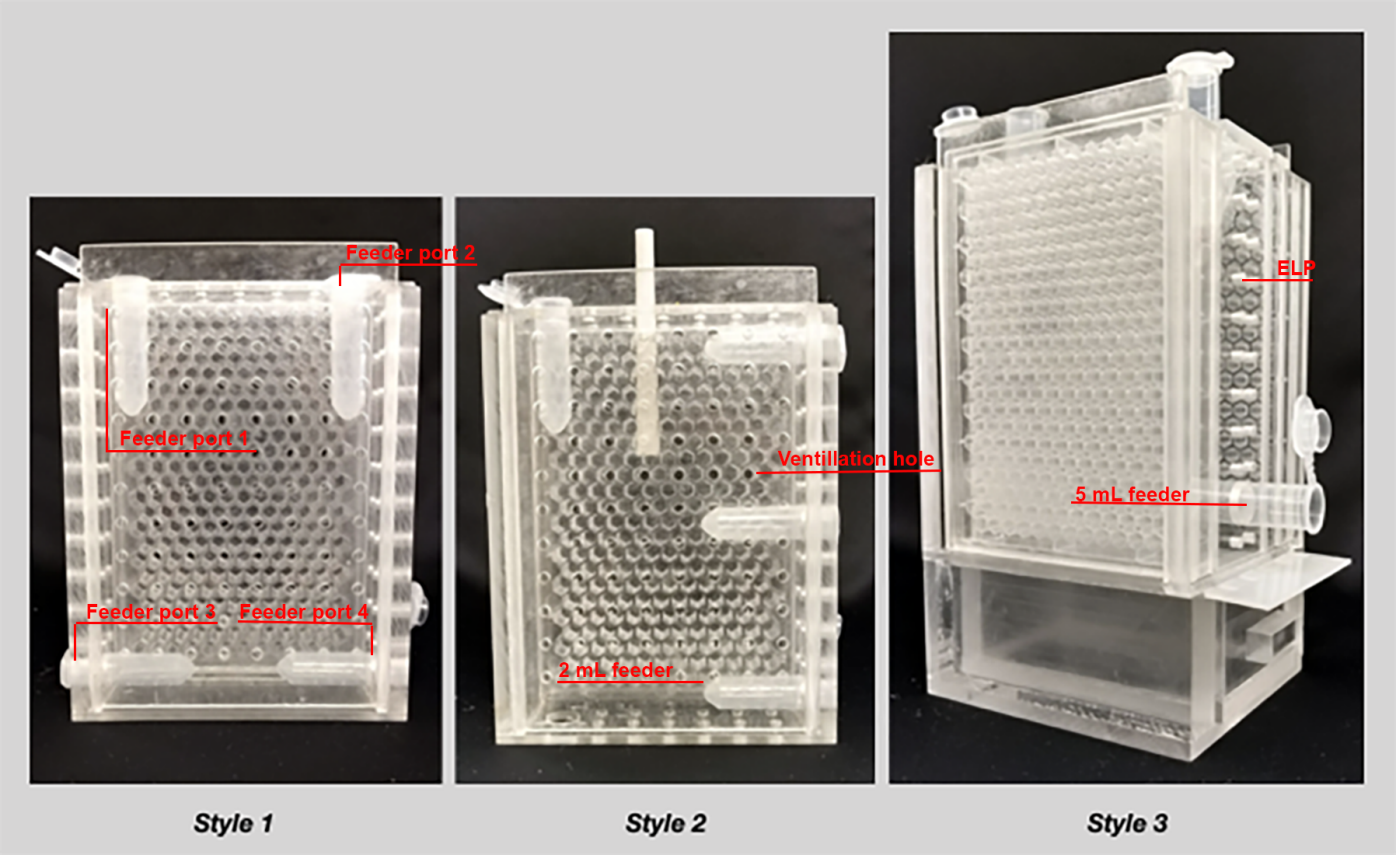
Queen monitoring cages (QMCs) styles 1, 2, and 3.

QMCs were composed of plexiglass with small holes in the walls for ventilation (Fig 1). Each QMC contained 1–2 egg laying plates (ELP) positioned vertically and serving as the inner walls of the cages. These custom made injection-molded, polystyrene plates were patterned with 264 hexagonal wells measuring 5.1 mm across and 11 mm deep, mimicking the dimensions of the cells in natural honey bee brood comb [47]. We chose to develop a system that does not require beeswax substrates because most samples of beeswax are contaminated with various agrochemical residues [12]. New ELPs were used for each experiment. Each QMC has four ports through which feeders containing pollen, sucrose solution, water, and honey can be inserted.

Three cage designs were used throughout our experiments to facilitate different experimental designs involving larger numbers of bees, and to explore how subtly different designs can facilitate the monitoring process.

#### Style 1

Style 1 is 8.3 cm X 2.8 cm X 12.3 cm (interior). A removable ELP is inserted into the back with a plexiglass insert behind it to block the bees from exiting the cage when the ELP is emptied or replaced. This QMC has four holes, each large enough to accommodate 2ml feeders.

#### Style 2

Style 2 has the same dimensions as QMC Style 1, however, in place of a plexiglass insert, a plastic adapter was placed between the interior of the cage and the ELP. The adapter is a 3-D printed (Viper SI, 3D Systems Inc., material: WaterClear Ultra 10122) outline of the 264 cells and provides an interface between the bees and the ELP. This allows for the ELP to be easily removed without disturbing the bees. A flexible plastic film inserted between the adaptor and the ELP is used to keep the bees from exiting the cage while the ELP is emptied or replaced. These adapters tend to warp during cleaning, therefore care must be taken to ensure their continued utility.

#### Style 3

Style 3 measures 8.3 cm X 4.5 cm X 12.3 cm (interior) with a removable drawer for the introduction and removal of workers. This QMC has four holes large enough to accommodate 5 mL feeders and incorporates two parallel ELPs that face each other. Plexiglass inserts are used to prevent bees from exiting the cage when ELPs are emptied or replaced.

Styles 1 and 2 were used in Experiment 1, with an equal number of both styles used in each treatment, and Style 3 was used in Experiments 2 and 3. All 3 cage styles performed well in these experiments, though Style 3 allowed for more bees to be used, facilitating the sampling of adult bees throughout experiments.

### Diets

Bee bread was collected from colonies by placing frames of empty wax comb in the center of the brood nest for three days. They were then removed and the bee bread was harvested from the wax comb cells. Fresh bee bread (BB) was fed to the caged bees after it was harvested without having been subjected to temperatures below 20°C. Bee bread from the same frames was also harvested and stored in a freezer at -80°C for at least 1 h before being thawed and fed to the caged bees (FBB) in order to test whether freezing could be implemented as a viable storage option for freshly collected bee bread. All of the bee bread in these experiments was stored in the colony for approximately 72 h based on research showing that nurse bees prefer freshly stored bee bread [40]. See S1 Supporting Text for further details on bee bread collections.

Commercial pollen was purchased from Betterbee Bee Supply (Greenwich, NY). Pollen paste diets were made using ground, commercial pollen stored at -20°C. The diets were made less than one hour prior to use according to the following recipe:

45% pollen paste (PP-45)– 45% commercial pollen, 35% local honey, 20% sucrose solution (30% w/v).

70% pollen paste (PP-70)– 70% commercial pollen, 30% local honey.

Percentages were based on weight.

In addition to pollen diet, QMCs were supplied with feeders containing honey, water, and 30% sucrose solution, each administered in 2 mL or 5 mL feeders.

### Incubator

QMCs were maintained in a Percival incubator with stable environmental conditions of 34°±0.5°C and 60%±10% relative humidity (RH), similar to the conditions inside a normal colony [18].

### Experiment 1: Effects of fresh bee bread and 45% pollen paste on egg laying

Twenty cages were assembled on July 19–20, 2017, each containing 100 newly eclosed worker bees and a queen. Ten of these cages were provisioned with bee bread (BB), and 10 were provisioned with 45% commercial pollen paste (PP-45). Eggs were counted twice daily between 9–11:00 and again between 18–20:00. After counting, the eggs were removed from the ELPs, which were then reinserted to the QMCs. Pollen diet consumption was measured every 2 days by removing feeders and recording the change in weight. Pollen feeders were replaced every 2 days with feeders containing freshly collected or prepared diet according to treatment. Egg laying was tracked in each cage for 13 days.

### Experiment 2: Effects of fresh bee bread vs. frozen bee bread vs. 45% pollen paste on egg laying

Forty-five QMCs were assembled on August 10–12, 2017, each containing 300 newly eclosed worker bees and a queen. Groups of 15 QMCs were each provisioned with either fresh bee bread (BB), frozen bee bread (FBB), or 45% commercial pollen paste (PP-45). BB and FBB were harvested from the same frames as described above. Egg laying and pollen consumption was monitored as in Experiment 1. Pollen feeders were replaced every 2 days with feeders containing freshly collected or prepared diet according to treatment, and 10 bees were removed through an empty feeder port using soft tweezers. The subsampled bees were flash-frozen in liquid nitrogen and stored at -80° C until they were dissected for HPG acinus measurement [48]. Egg laying was tracked in each cage for 14 days.

Bees subsampled on August 18^th^ from 39 of the QMCs (14 BB, 13 FBB, and 12 PP-45) were selected for HPG dissection and measurement of acinus size. HPG dissections were performed by first removing the bee heads over dry ice, and the exoskeleton was removed in ethanol chilled with dry ice. The heads were then transferred to room temperature ethanol, and the glands were removed using a pair of forceps under an Olympus Szx12 stereomicroscope. Morphological measurements of the acini were performed on stored images taken with the stereo microscope as described by Hrassnigg et al. [48]. The average width (μm) of 10 acini from each bee was measured using the straight-line tool in ImageJ [49].

### Experiment 3: Effects of frozen bee bread vs. 70% pollen paste on egg laying

Thirty cages were assembled on October 14, 2017, each containing 200 newly eclosed worker bees and a queen. Fifteen of the cages were provisioned with bee bread stored at -80°C (FBB) and the other 15 were provisioned with 70% commercial pollen paste (PP-70). Egg laying and pollen consumption was monitored as in Experiments 1 and 2. Pollen feeders were replaced every 2 days with feeders containing fresh diet. Egg laying was monitored and recorded daily for 10 days. The cages were disassembled on the 11^th^ day due to observations of heavy mortality of worker bees. A smaller number of worker bees were added to each cage in this experiment because we did not sample worker bees.

### Statistical analyses

A Student’s *t*-test or one-way ANOVA with a post hoc Tukey’s Honest Significant Difference test (Tukey HSD) was used to evaluate the effects of diet treatments on the total number of eggs laid in each experiment (JMP Pro 12). In Experiment 1, the cage styles were distributed equally across treatment, therefore we did not need to account for cage style in the analysis of treatment effects.

Poisson loglinear generalized estimating equations (GEE) with autoregressive (AR-1) correlation matrices (IBM SPSS Statistics 24) were used to assess the effects of pollen diet on egg laying across time, with each day and treatment group treated as factors and each queen treated as a subject effect with day as a within subject effect. We cannot confidently treat queen egg laying on a given day as independent from egg laying on an adjacent day, therefore the AR-1 correlation matrix structure was conservatively selected instead of an independent correlation matrix structure based on its appropriateness for data sets with a high number of factors that are not independent. GEE analysis accounts for within-subject variation and does not exclude subjects with incomplete datasets (as in the case of a queen death), and the GEE β-coefficients can be used to estimate the magnitude and direction of significant effects relative to a reference [50]. In our experiments, we used the treatment groups with the lowest average egg laying as the reference treatments (PP-45, PP-45, and PP-70 in Experiments 1, 2, and 3, respectively), and the reference time points were the final day of each experiment. A Wald Chi-Square post hoc test was used to determine the significance of treatments (when more than 2 were compared), each day, and each interaction term. A Bonferroni correction for multiple comparisons was applied to p-values when evaluating the effects of time (day) and interaction effects. In Experiment 2, no egg laying was observed in one or more treatments on the first 2 days of the experiment. To conform to the assumptions of the GEE analysis, these days were excluded from the GEE. In Experiment 3, no egg laying was observed in either treatment for the first 2 days of the experiment. These days were excluded from the GEE and additionally were not used to calculate average daily egg production.

To identify differences in pollen consumption between treatment groups, Wilcoxon Rank Sum tests and Kruskal Wallis tests were performed in JMP Pro 12. Correlations between time and pollen consumption were assessed using Spearman’s ρ, estimated using JMP Pro 12. Significance was evaluated at a 0.05 level, and 0.1 was considered evidence of a trend. All experimental data are available in S1 File.

## Results

Throughout our experiments, most queens in all treatment groups laid eggs in QMCs. See S1 Table for a summary of egg laying observed in all experiments. Variation in the onset of egg laying was observed during these experiments, with some queens beginning to lay after less than 24 hours and most laying within 72 hours. Because of this variation, an adaptation period during which the cages are not disturbed in the incubator may be considered for future experiments using this system.

During Experiment 1, one queen in the pollen paste treatment group died between the monitoring periods on day 6 and 7, and no data from this QMC after day 6 were used in any statistical analysis. No queen mortality was observed in Experiments 2 and 3.

### Experiment 1: Effects of bee bread vs. 45% pollen paste on egg laying

**Fig 2:**
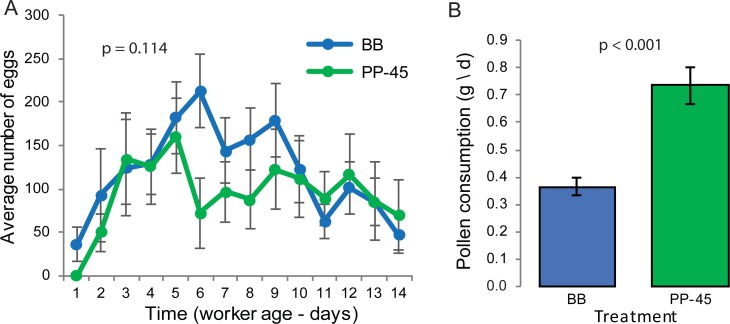
Effects of pollen diets on egg laying and pollen diet consumption (Experiment 1). **A.** Average ± SE number of eggs laid per day in QMCs provisioned with bee bread (BB, blue) or 45% pollen paste (PP-45, green). The p-value is the result of GEE analysis (p = 0.114, Wald Chi-Square = 2.3, df = 1). **B.** Average ± SE pollen diet consumed per day in BB (blue) and PP-45 (green) treatment groups. The p-value is the result of a Wilcoxon Rank Sum test (p<0.001, Chi-Square = 18.7, df = 1).

There was no significant difference in total eggs laid by queens in QMCs provisioned with BB vs. PP-45 (p = 0.45, Student’s t-test, t = -0.8, df = 18), and there was no significant difference in daily egg laying between QMCs provisioned with BB vs. PP-45 (p = 0.114, GEE, Wald Chi-Square = 2.3, df = 1, Fig 2A). Egg laying changed over time, with a statistically significant effect of time on egg laying (p<0.001, GEE, Wald Chi-Square = 1818.6, df = 13). Egg laying on day 1 was significantly lower than egg laying on the final day (Table 1). A significant interaction was detected between time and treatment (p<0.001, GEE, Wald Chi-Square = 280.5, df = 13). This interaction was evident on day 1 (Table 2). The positive β coefficient indicates that the increase in egg laying due to BB diet was greater on day 1 than on day 14.

**Table 1:** Effects of worker age on queen egg laying (GEE, Wald Chi-Square post hoc test). Directionality and magnitude can be interpreted from the β-coefficient with reference to the final time point (Day).

Experiment	Day	β-coefficient	Wald Chi-Square	p-value	Bonferroni adj. p-value
**1**	**1**	5.8±1.2	24.5	<0.001	<0.001
**2**	**10**	-1.7±0.4	14.5	<0.001	0.002
**3**	**3**	-4.0±0.7	34	<0.001	<0.001
	**4**	-1.6±0.4	16.5	<0.001	<0.001
	**5**	-0.6±0.2	6.3	0.012	0.084
	**7**	0.6±0.2	14.3	<0.001	0.001
	**8**	0.4±0.2	4.7	0.03	0.21
	**9**	0.3±0.1	5	0.03	0.182

**Table 2:** Significant interaction effects of pollen diet and time on egg laying (GEE, Wald Chi-Square post hoc test). Directionality and magnitude can be interpreted from the β-coefficient with reference to PP-45 (Exp. 1 and 2) or PP-70 (Exp. 2) and the final time point (Day).

Experiment	Day	Treatments compared	β-coefficient	Wald Chi- Square	p-value	Bonerroni adj. p-value
**1**	**1**	**BB vs. PP-45**	-5.6±1.3	17.7	<0.001	<0.001
**2**	**10**	**BB vs. PP-45**	1.8±0.5	15.2	<0.001	0.002
	**3**	**FBB vs. PP-45**	-1.7±0.5	12.1	0.001	0.024
	**4**	**FBB vs. PP-45**	-1.0±0.4	6.7	0.009	0.198
	**5**	**FBB vs. PP-45**	-1.0±0.4	5.7	0.017	0.374
	**6**	**FBB vs. PP-45**	-0.8±0.3	9.6	0.002	0.044
	**7**	**FBB vs. PP-45**	-0.8±0.2	13.5	<0.001	0.005
	**10**	**FBB vs. PP-45**	1.1±0.5	5.7	0.017	0.374
**3**	**3**	**FBB vs. PP-70**	2.4±0.8	9.8	0.002	0.014
	**4**	**FBB vs. PP-70**	1.2±0.5	6.5	0.011	0.077
	**5**	**FBB vs. PP-70**	0.7±0.3	5.8	0.016	0.112

Significantly greater amounts of pollen diet were consumed by bees in QMCs fed PP-45 relative to BB (p<0.001, Wilcoxon Rank Sum, Chi-Square = 18.7, df = 1, Fig 2B). A significant negative correlation was detected between time and pollen consumption (p≤0.0001, Spearman’s ρ = -0.8).

### Experiment 2: Effects of fresh bee bread vs. frozen bee bread vs. 45% pollen paste on egg laying

**Fig 3:**
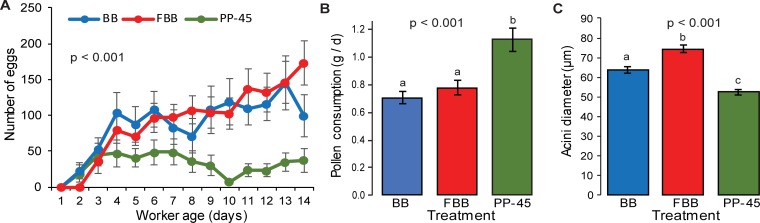
Effects of pollen diets on egg laying, pollen diet consumption and hypopharyngeal gland acinus diameter (Experiment 2). **A.** Average ± SE number of eggs laid per day in QMCs provisioned with bee bread (BB, blue); frozen bee bread (FBB, red) or with 45% pollen paste (PP-45, green). The p-value is the result of GEE analysis (p = 0.001, Wald Chi-Square = 13.8, df = 2). **B.** Average ± SE pollen diet consumed per day in BB (blue); FBB (red) and PP-45 (green) treatment groups. The p-value is the result of a Kruskal Wallis test (p = 0.0006, Chi-Square = 14.9). **C.** Average ± SE acini diameter of worker bees sampled on day 7 and 8 from QMCs provisioned with BB (blue), FBB (red), and PP-45 (green). The p-value is the result of a Kruskal Wallis test (p<0.0001, Chi-Square = 50.0).

A significant effect of diet on total eggs laid by queens was observed (p = 0.01, ANOVA, F = 5.1, df = 2), and queens in PP-45 provisioned QMCs laid fewer eggs than queens in BB or FBB provisioned QMCs (Tukey HSD). A significant effect of pollen diet type on daily egg laying was observed (p = 0.001, GEE, Wald Chi-Square = 13.8, df = 2), with queens in FBB QMCs laying significantly more eggs per day than queens in PP-45 QMCs (p = 0.001, Wald Chi-Square = 11.4, df = 1, Fig 3A). A trend was observed for queens in BB QMCs to lay more eggs on a daily basis than PP-45 QMCs (p = 0.06, Wald Chi-Square = 3.7, df = 1, Fig 3A). Time significantly affected egg laying (p<0.001, GEE, Wald Chi-Square = 63.5, df = 11). Significantly lower egg laying was observed on day 10 relative to the final time point (Table 1).

A significant interaction was detected between time and treatment (p<0.001, Wald Chi-Square = 174.6, GEE, df = 22). This interaction was observed in QMCs provisioned with BB relative to PP-45 on day 10. The positive β coefficient of the interaction term indicates that the increase in egg laying related to BB treatment was greater on day 10 relative to the increase at the final time point. This is explained by a decrease in the number of eggs laid in PP-45 QMCs but not in BB QMCs on day 10. In QMCs provisioned with FBB, 6 time points are affected by an interaction between time and treatment, but only 3, days 3, 6, and 7 were significant after a Bonferroni p-value correction for multiple tests. On these days, the negative β coefficients of the interaction terms indicate that the increases in egg laying related to FBB treatment was less than the increase at the final time point. This is explained by continuing increase in average eggs laid per day in FBB provisioned cages until the conclusion of the experiment. See Table 2 for a summary of the affected time points by treatment comparisons.

Diet type significantly affected pollen consumption in QMCs (p = 0.0006, Kruskal Wallis test, Chi-Square = 14.9). Significantly greater amounts of pollen diet were consumed by bees in QMCs provisioned with PP-45 relative to BB or FBB (BB: p = 0.0002, Wilcoxon Rank Sum, Chi-Square = 13.7, df = 1; FBB: p = 0.005, Chi-Square = 8.0, df = 1, Fig 3B). No differences in pollen consumption were detected between QMCs provisioned with BB or FBB (p = 0.4, Wilcoxon Rank Sum, Chi-Square = 0.7, df = 1). A significant negative correlation was detected between time and pollen consumption (p≤0.0001, Spearman’s ρ = -0.7).

Diet type had a significant effect on the average acini width in worker bees sampled on day 7 or 8 of this experiment (p<0.0001, Kruskal Wallis test, Chi-Square = 50.0). The average acini diameters were significantly different among the 3 treatments (BB vs. FBB: p = 0.006, Wilcoxon Rank Sum, Chi-Square = 7.5, df = 1; BB vs. PP-45: p≤0.0001, Chi-Square = 19.7, df = 1; FBB vs. PP-45: p≤0.0001, Chi-Square = 48.5, df = 1, Fig 3C). The bees in QMCs provisioned with FBB had the largest HPG acini, followed by BB and PP-45, in that order.

### Experiment 3: Effects of frozen bee bread vs. 70% pollen paste on egg laying

**Fig 4:**
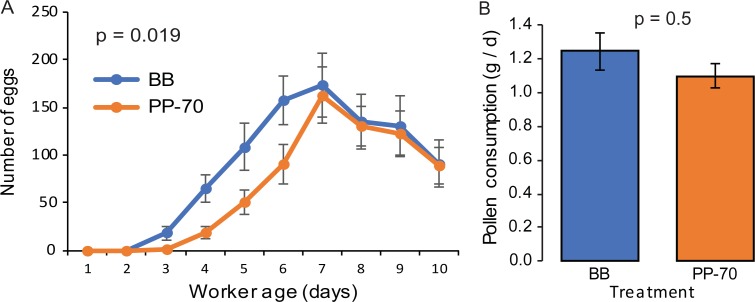
Effects of pollen diets on egg laying and pollen diet consumption (Experiment 3). **A.** Average ± SE number of eggs laid per day in QMCs provisioned with bee bread (BB, blue) or 70% pollen paste (PP-70, orange). The p-value is the result of GEE analysis (p = 0.019, Wald Chi-Square = 5.5, df = 1). **B.** Average ± SE pollen diet consumed per day in BB (blue) and PP-70 (orange) treatment groups. The p-value is the result of a Wilcoxon Rank Sum test (p = 0.5, Chi-Square = 0.4, df = 1).

No significant effect of pollen diet on total eggs laid was observed (p = 0.3, Student’s *t*-test, *t* = -1.1, df = 28). However, as in Experiment 2, there was a significant effect of diet type on daily egg laying (p = 0.019, GEE, Wald Chi-Square = 5.5, df = 1, Fig 4A). Time significantly affected egg laying (p<0.001, GEE, Wald Chi-Square = 204.8, df = 7). Significantly lower egg laying was observed on the days 3 and 4 relative to the final time point after Bonferroni correction, but significantly higher egg laying was observed on day 7, indicating that peak performance was achieved at this time (Table 1).

A significant interaction was detected between time and treatment (p≤0.005, GEE, Wald Chi-Square = 20.1, df = 7). The interaction was evident on days 3, 4, and 5, but only day 3 was significant after Bonferroni adjustment. The positive β coefficient of this interaction indicates that the increase in egg laying related to BB treatment was greater on day 3 relative to the final time point (Table 2).

No significant difference in pollen consumption was detected between treatments (p = 0.5, Wilcoxon Rank Sum, Chi-Square = 0.4, df = 1, Fig 4B). A significant negative correlation was detected between time and pollen consumption (p≤0.0001, Spearman’s ρ = -0.7).

## Discussion

We have presented a new method to track and quantify queen egg laying under controlled laboratory conditions and used it to test the effect of pollen diet on egg laying. The use of Queen Monitoring Cages (QMCs) allowed us to examine the relationship between worker diet and physiology and queen fecundity, yielding robust data from a large sample size. This versatile system could be adapted for many purposes including honey bee risk assessment and egg collection to produce transgenic bees [51].

During these experiments, the number of eggs laid did not reach the high end for queens in full sized colonies reported in the literature [18], suggesting that further manipulations can increase egg production in this system. However, the majority of queens readily laid eggs in QMCs and responded to diet treatments, suggesting that the system is also suitable for risk assessment experiments.

The results of Experiments 2 and 3 indicate that provisioning QMCs with bee bread can positively influence queen egg laying. This difference was particularly striking in Experiment 2, when provisioning QMCs with frozen bee bread resulted in nearly 3 times more eggs than 45% pollen paste. This may be due in part to the differences in pollen composition of the diets. Percentages of pollen in bee bread as high as 88% have been reported [52], and, as the primary source of protein, lipids, and many vitamins and minerals for honey bees, pollen is essential to the health of a colony [36,53]. The relatively higher consumption of pollen paste in Experiment 2 may have been a compensatory response to the lower percentage of pollen relative to the other diets. This is consistent with the finding that when the percentage of pollen in pollen paste was increased to 70%, no difference could be detected in total eggs laid, and a much smaller disparity in the daily number eggs laid between bee bread and pollen paste-provisioned QMCs that diminished over time was observed.

Bee bread was also shown to positively affect the size of worker bee hypopharyngeal glands (HPGs), suggesting a mechanism for the effect of worker nutrition on queen egg laying. Within a colony, the queen receives her nutrition through trophallaxis from young workers who form a retinue around her [18]. Although a direct relationship between HPG development and queen retinue behaviors has not been established, the results of Experiment 2 strongly suggest that worker HPG development influences queen egg laying productivity. This may be directly related to the ability of worker bees to provision the queen with proteinaceous secretions produced by the HPGs [25,36]. In other insect species it is well known that reproduction is heavily dependent on individual nutrition [54], but in these experiments, the pollen diet was not directly consumed by the queen. These results suggest that the egg laying of the honey bee queen is dependent on worker nutrition, providing another example by which the colony functions as a superorganism[55].

Average HPG acinus diameter in bees from QMCs provisioned with bee bread was still smaller than what has been reported in the literature for similarly aged bees [48]. Perhaps this is because at the time the bees were sampled, egg laying had not yet peaked, and worker HPG development also had not yet peaked. An alternative explanation is that because QMCs were populated only with younger bees, some bees may have experienced accelerated development resulting in more forager-like physiology, with smaller HPGs. This phenomenon is based on social inhibition of adult maturation and has been previously reported in single-cohort colonies initially composed of all young worker bees [56,57].

Although there were no differences in egg laying between queens in QMCs provisioned with bee bread or frozen bee bread, workers from QMCs provisioned with frozen bee bread had higher average HPG gland sizes. This may be because freezing plant material degrades the cell wall components [58], potentially making pollen easier to digest. Our results demonstrate that freezing bee bread at -80°C is an acceptable form of short term storage and may even contribute to successful egg laying in QMCs. The duration and conditions of pollen storage are known to affect its quality and suitability for brood rearing [59], therefore more research is needed to determine if bee bread can be stored in this manner for longer periods of time. Additionally, more research is needed to determine what components of bee bread contribute positively to egg laying.

In all experiments, egg laying rates were low initially and increased over the first few days. This may be because the bees require time to adjust to the cage after their introduction, but another compelling hypothesis is that worker age has an effect on queen egg laying in QMCs. This would not be surprising, as worker honey bees exhibit striking patterns of physiological and behavioral maturation, a function of the colony’s age-related division of labor [60]. This also has been observed in laboratory cages [61], and was likely the cause of the patterns observed here. This may explain the time by treatment interaction effects on queen egg laying seen in Experiments 2 and 3. This interaction was particularly strong in Experiment 2, where we observed egg laying continuing to increase until the final time point in bee bread provisioned QMCs relative to pollen paste.

Variation in egg laying between experiments suggests that factors external to this study can influence egg laying in QMCs. Overall, the highest egg laying was observed in Experiment 1, which was conducted in July. Experiment 2 was conducted less than 1 month later, but much lower rates of egg laying were observed in cages fed PP-45 relative to BB and FBB. In Experiment 3, higher rates of egg laying were evident in BB fed cages relative to PP-70, but only during the first half of the experiment. Worker physiology varies with season [62–66] and with conditions during pre-adult development [67,68]. The workers used in Experiment 1 may have been better suited for the tasks associated with supporting queen egg laying due to physiological differences caused by either of these factors. Additionally, pathogen abundance within colonies can change over time, often increasing throughout the active season [69]. It is not clear how pathogen infections would affect egg laying in QMCs, but behavioral effects associated with viral infections, including learning deficits and increased aggression, have been documented [70,71], suggesting that pathogen infections would have a negative effect on egg production in QMCs. One interpretation of the results of all 3 experiments taken together is that diet can buffer the effects of developmental background on worker physiology, better equipping them to care for and provision the queen.

Other sources of variation that may have contributed to the differences in results between experiments and that would be worth considering in the future are seasonal variation in bee bread composition and the effects of worker honey bee genetic variation. A recent study by Degrandi-Hoffman et al. [72] found that gene expression profiles in honey bee fat bodies vary with the time of year and the seasonality of their pollen diet, suggesting that bees in QMCs can also be manipulated this way in the future. Additionally, genetic variation among workers has been shown to affect virtually every trait studied, at the molecular, physiological, and behavioral levels [73,74], so it is possible that there also is variation for physiological and behavioral traits that affect queen egg laying.

One potential application of the QMC system is the collection of honey bee embryos for genome editing via CRISPR/Cas9 [75]. Because of the fragility of honey bee eggs, special care must be taken when manipulating them, and eggs are typically taken directly from full sized colonies for these purposes using a specialized queen cage (Jenter Queen Rearing Kit) [76]. Although we did not collect eggs during these experiments, this system could be easily adapted for this purpose. CRISPR/Cas9 has been used successfully in four hymenopteran species including honey bees [51,77–80], and its utility in studying the roles of specific genes in social evolution has already been demonstrated [77,78]. In the future, gene editing techniques could be applied to honey bee embryos collected using QMCs to further our understanding of honey bee biology, health, and responses to stress.

There is currently a pressing need to develop systems to examine and quantify the effects of single and multiple stressors on honey bee queen health. Queen egg laying is affected by a variety of seasonal, nutritional and social factors [10,18,25,26], and research suggests it may be vulnerable to disruption via these stressors [21,23,24,30,81]. In the future, the system presented here should be used for experiments to assess the single and combined effects of pesticides, pathogens, parasites and nutrition on egg laying. Paired with extant and additional field study data, the findings of experiments performed with QMCs could greatly aid in predicting, assessing, and mitigating health risks to the honey bee population and pollination services.

## Supporting information

S1 Supporting Text Bee bread collection. (DOCX)

S1 File Egg laying data. (XLSX)

S1 Table Average eggs laid per day, maximum eggs laid per day, and laying vs. non-laying queens by experiment and treatment. (DOCX)
