# Supplementary material for: Quantifying the effects of pollen nutrition on honey bee queen egg laying with a new laboratory system
Source: PLoS One. 2018 Sep 5;13(9):e0203444. doi: 10.1371/journal.pone.0203444 (PMC6124782; doi:10.1371/journal.pone.0203444)
Supplement: S1 Supporting Text — (DOCX) [file pone.0203444.s001.docx]

**S1 Supporting Text**

**Bee Bread Collection**

**Bee Bread in Experiment 1**: Bee bread was collected from colonies maintained by the Honey Bee Research Facility at the University of Illinois at Urbana-Champaign. Frames of empty drawn comb were placed in the center of the brood nest of strong colonies and left for one to three days. They were then removed and the bee bread was harvested from the cells. Bee bread was collected from frames immediately after they were removed from hives and used to refill bee bread feeders. After harvesting, frames were replaced with empty frames which were collected for use every other day.

**Bee Bread in Experiment 2**: Bee bread was frequently collected for this experiment as in experiment 1. However, to compensate for decreased pollen foraging, bee bread was occasionally harvested from a frame over the course of several days. During this time, the frame was kept indoors at room temperature.

**Frozen Bee Bread in Experiment 3**: The bee bread used in experiment 3 was collected during Experiments 1 and 2 and stored in the -80C freezer. Additional bee bread was also harvested from colony frames as needed and frozen.
