# Supplementary material for: Quantifying the effects of pollen nutrition on honey bee queen egg laying with a new laboratory system
Source: PLoS One. 2018 Sep 5;13(9):e0203444. doi: 10.1371/journal.pone.0203444 (PMC6124782; doi:10.1371/journal.pone.0203444)
Supplement: S1 Table — (DOCX) [file pone.0203444.s003.docx]

**S1 Table: Average eggs laid per day, maximum eggs laid per day, and laying vs. non-laying queens by experiment and treatment.**

|  |  | **Average eggs laid in 24 hours ± SE** | **Maximum eggs laid in 24 hours** | **Laying queens/Non-laying queens** |
| --- | --- | --- | --- | --- |
| **Experiment 1** | **Bee Bread** | 119.7 ± 10 | 456 | 10/0 |
|  | **45% Pollen Paste** | 93.7 ± 11 | 408 | 8/2 |
| **Experiment 2** | **Bee Bread** | 94 ± 7.4 | 434 | 15/0 |
|  | **Frozen Bee Bread** | 99 ± 6.6 | 378 | 14/1 |
|  | **45% Pollen Paste** | 34 ± 3.9 | 246 | 13/2 |
| **Experiment 3** | **Frozen Bee Bread** | 110 ± 9.7 | 355 | 14/1 |
|  | **70% Pollen Paste** | 83.4 ± 8.1 | 395 | 15/0 |
